# Is histogram manipulation always beneficial when trying to improve model performance across devices? Experiments using a Meibomian gland segmentation model

**DOI:** 10.3389/fcell.2022.1067914

**Published:** 2022-12-05

**Authors:** Xianyu Deng, Lei Tian, Yinghuai Zhang, Ao Li, Shangyu Cai, Yongjin Zhou, Ying Jie

**Affiliations:** ^1^ Health Science Center, School of Biomedical Engineering, Shenzhen University, Shenzhen, China; ^2^ Marshall Laboratory of Biomedical Engineering, Shenzhen, China; ^3^ Beijing Ophthalmology and Visual Sciences Key Laboratory, Beijing Tongren Eye Center, Beijing Tongren Hospital, Beijing Institute of Ophthalmology, Capital Medical University, Beijing, China; ^4^ Ophthalmology and Visual Sciences Key Laboratory, Beijing, China

**Keywords:** Meibomian gland, segmentation, histogram, image, model performance

## Abstract

Meibomian gland dysfunction (MGD) is caused by abnormalities of the meibomian glands (MG) and is one of the causes of evaporative dry eye (DED). Precise MG segmentation is crucial for MGD-related DED diagnosis because the morphological parameters of MG are of importance. Deep learning has achieved state-of-the-art performance in medical image segmentation tasks, especially when training and test data come from the same distribution. But in practice, MG images can be acquired from different devices or hospitals. When testing image data from different distributions, deep learning models that have been trained on a specific distribution are prone to poor performance. Histogram specification (HS) has been reported as an effective method for contrast enhancement and improving model performance on images of different modalities. Additionally, contrast limited adaptive histogram equalization (CLAHE) will be used as a preprocessing method to enhance the contrast of MG images. In this study, we developed and evaluated the automatic segmentation method of the eyelid area and the MG area based on CNN and automatically calculated MG loss rate. This method is evaluated in the internal and external testing sets from two meibography devices. In addition, to assess whether HS and CLAHE improve segmentation results, we trained the network model using images from one device (internal testing set) and tested on images from another device (external testing set). High DSC (0.84 for MG region, 0.92 for eyelid region) for the internal test set was obtained, while for the external testing set, lower DSC (0.69–0.71 for MG region, 0.89–0.91 for eyelid region) was obtained. Also, HS and CLAHE were reported to have no statistical improvement in the segmentation results of MG in this experiment.

## Introduction

Meibomian glands (MG) are special sebaceous gland located on the tarsal plate of eye (Erich et al., 2011). They produce meibum to prevent tear from over evaporation (Nichols et al., 2011), maintain the surface tension of the tear film, and trap tears between its oily edge and the eyeball. Healthy MG are elongated in shape, arrange in parallel and throughout the entire tarsal plate (Djalilian, 2018). Functional and/or structural problem of MG may cause meibomian gland dysfunction (MGD) (Driver and Lemp, 1996). MGD is a chronic, diffuse abnormality of the meibomian glands, commonly characterized by terminal duct obstruction and/or qualitative/quantitative changes in the glandular secretion (Den et al., 2011; Nichols et al., 2011). MGD often leads to tear film alteration, ocular surface disease, and is the leading cause of evaporative dry eye (Turgut et al., 2018), which can seriously affect the patient’s life and increase the global public health and financial burden (McDonald et al., 2016).

Meibomian gland area loss is an important index to evaluate MGD (Arita et al., 2014). MGD can be directly observed using meibography, which is an optical imaging technique allowing visualizing MG morphology *in vivo* (Pult and Nichols, 2012). Recently, many non-contact infrared meibography methods were developed, making the MGD diagnosis process more patient-friendly and less time-consuming (Pult and Riede-Pult, 2012; Wong et al., 2019; Xiao et al., 2020; Hwang et al., 2021). These devices allow users to capture high-resolution images of meibomian glands in a short period of time, which can provide sufficient experimental material for MG analysis. Quantification of the area of meibomian glands loss is of importance when assessing MGD. To date, automatic methods based on image processing techniques have been developed for the automated assessment and classification of MGD (Arita et al., 2014; Celik et al., 2013; Koprowski et al., 2016; Koprowski et al., 2017; Liang et al., 2017; Llorens-Quintana et al., 2019; Pult and Riede-Pult, 2013; Xiao et al., 2021). In recent years, MGD automatic analysis methods based on convolutional neural network (CNN) have been developed rapidly. These works automatically segment the eyelid and MG, and calculate the loss rate and analyze the morphological parameters of MG (Wang et al., 2019). In order to promote the segmentation performance of MG, contrast limited adaptive histogram equalization (CLAHE) will be used as an image preprocessing step to enhance the contrast of MG images (Prabhu et al., 2020; Dai et al., 2021).

However, the MG images used in these works were acquired from a single device. The preset parameters of the method or the trained model are for the specific data domain used in the experiment. When testing these methods with images from other distribution domains (such as different image modalities, or different acquisition devices), a lower performance is usually obtained (Yan et al., 2019). Such problem is called “distribution shift” (Jo and Bengio, 2017). To reduce the performance gap, an effective method is to reduce the distribution domain distance of the data. Training a generative adversarial network (GAN) to generate fake images between two different domains is a common method to improve the generalization of the model to data from different domains (Perone et al., 2019; Yan et al., 2019); but it requires a large number of training samples. In some medical image processing tasks, histogram specification (HS) has been reported as an effective method for contrast enhancement and improving model performance on images of different modalities, such as between MRI and CT images (Naseem et al., 2021).

In this study, we focus on two open questions in the field of automated meibomian gland analysis:

1) In the CNN-based MG analysis method, does CLAHE improve the network performance?
2) Can HS improve the performance of models trained with data collected from a single device on cross-device data?

This study aims at developing and validating an MG segmentation method based on CNN. Whether HS and CLAHE as preprocessing methods can effectively improve the segmentation performance of the CNN model was also investigated in this study. MG images captured from different devices were divided into different testing sets based on different preprocessing methods, and then segmented by CNN model to calculate MG loss rate. All the results will be compared with the ground-truth by the clinicians.

## Related works

Histogram specification (HS) enhances the brightness and contrast of the input image and transforms the input image into an image with a similar shape to the template histogram. HS has the advantage of simplicity and low computational cost (Xiao et al., 2018). HS is very common in the preprocessing stage of medical images. HS is used to match the histogram of the input image to the template image to initialize and avoid gradient explosion before using CNN to segment the kidney based on CT images (da Cruz et al., 2020). Naseem et al. (2021) based on HS to enhance images of different modalities. They enhance low-contrast CT images based on MRI images based on the second-order distribution.

The raw MG image often has low contrast. In other word, the pixel intensity of glands and background have little difference, as a result, it is difficult for the observer to separate the glands from background, especially when the image is blur. CLAHE is applied for the meibography image enhancement. CLAHE enhances an image by limiting the height of the local region histogram, such as a 64 pixels neighborhood block, thereby overcome the global uneven illumination and noise amplification problem. CLAHE is often used as a preprocessing method to enhance the performance of MG segmentation (Prabhu et al., 2020; Dai et al., 2021).

## Materials

This study involves 287 subjects (age: 56.1 ± 17.2 years old, 83 men and 204 women) collected by Beijing Tongren Hospital. The purpose and possible consequences of the study were explained to all involved subjects. Exclusion criteria included 1) ocular allergies, 2) history of ocular surgery, 3) history of ocular trauma, 4) other eye diseases, 5) long term or frequent contact lens use and 6) Any other eye or systemic disease known to affect the tear film. Excluded images include: 1) Images included something other than the eyelids and their surrounding tissueswere, 2) Images were not sufficiently clear for automatic analysis were excluded. 3) Patients’ eyes exhibited excessive meibomian lipid secretion. The study was approved by the Ethical Committee of the Beijing Tongren Hospital and was conducted in accordance with the tenets of the Declaration of Helsinki. A total of 1,074 images were collected by professional clinicians, including 888 MG images collected from Keratograph 5M (K5M; OCULUS Optikgeräte GmbH, Wetzlar, Germany) and 186 from KANGHUA DED-1L (KH). Note that $I_{k5m}$ is used to represent the image set collected from K5M, and $I_{kh}$ is used to represent the image collected from KH. Then, $I_{k5m}$ was divided into two sets, 648 for training set used for neural networks training, and 240 for testing set to appraise the algorithm performance. And $I_{kh}$ is used as an external testing set to test the performance of our models on data from different device. It should be noted that as a retrospective study, some of the 287 patients have received treatment and collected follow-up data at different times, therefore there are more meibomian gland images than the subject number.

**FIGURE 1:**
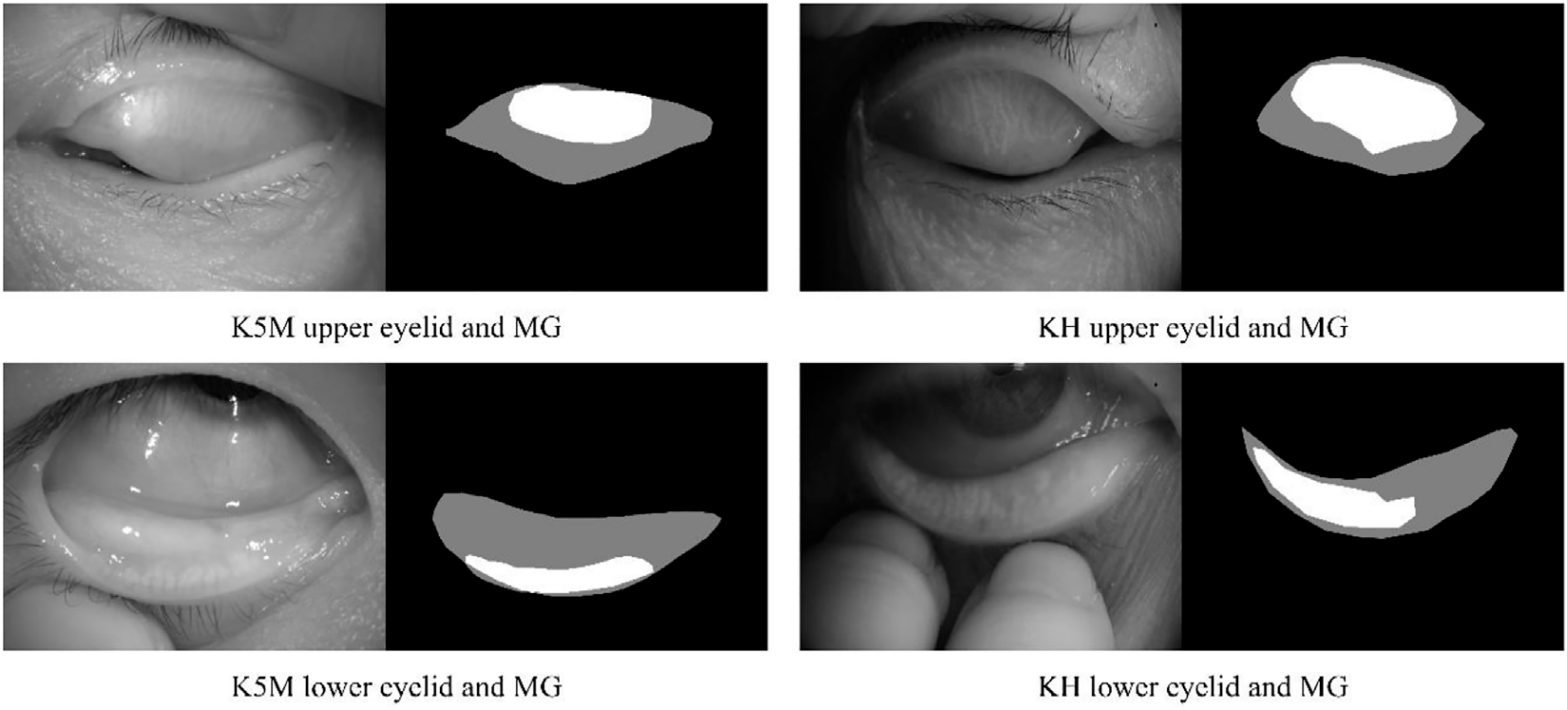
Examples of upper and lower eyelids and MG, and ground-truth for segmentation mask. Black, gray, and white pixel represent the background, eyelid region and MG region, respectively.

The ground-truth annotation was completed by a senior clinician using imageLabeler application in MATLAB (9.5.0.944444, R2018b, Java 1.8.0_152-b16). For labeling the eyelid region, the upper edge is defined at the opening of the gland, the lower edge is defined at the edge of proximal tarsal plate, and the horizontal borders is defined at the top and bottom borders intersected (Wang et al., 2019). Value 1 and 2 are used to denote the eyelid region and the MG region, respectively. Examples are shown in Figure 1.

## Methods

### Image preprocessing

Images $I_{kh}$ and $I_{k5m}$ acquired by two different devices are involved in this study. The grayscale histograms of the two image sets are significantly different, as shown in the figure. HS is applied to convert the grayscale density so that $I_{kh}$ can be matched to the histogram of $I_{k5m}$ . Specifically, the brightness distribution of each image in $I_{k5m}$ is counted, and the results are averaged to obtain an average histogram $H_{k5m}$ representing $I_{k5m}$ . Then, each image of $I_{kh}$ is matched to $H_{k5m}$ using HS, resulting in a similar pixel distribution to $I_{k5m}$ , as shown. $I_{kh-HS}$ is used to represent $I_{kh}$ after HS preprocessing. To explore the effect of CLAHE on segmentation performance, $I_{k5m}$ , $I_{kh}$ and $I_{kh-HS}$ were preprocessed with CLAHE. $I_{k5m-CLAHE}$ , $I_{kh-CLAHE}$ and $I_{kh-HS-CLAHE}$ are used to represent each set of images processed using CLAHE.

### MG segmentation network

**FIGURE 2:**
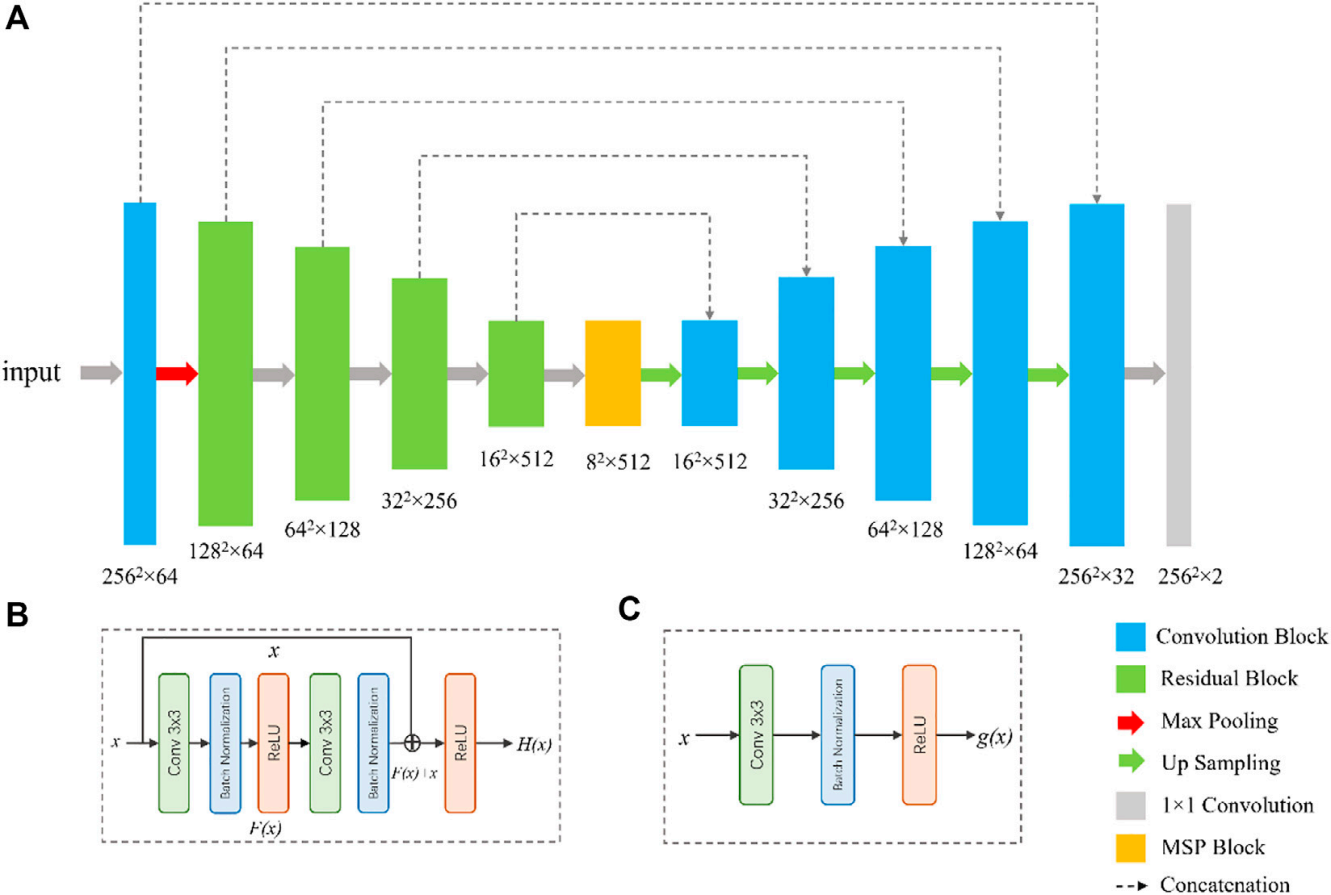
Network architecture for the segmentation of tear meniscus. The network **(A)** is a U-Net-like architecture with a modified ResNet-34 as encoder. The bottom of the network is multi-scale perception block (MSP). **(B)** and **(C)** are the illustrations of the residual block and convolution block, respectively.

To segment the eyelid region and MG region, we constructed a compact CNN based on U-Net architecture (Ronneberger et al., 2015). The segmentation network contains an encoder and a decoder, as shown in Figure. Specifically, we use ResNet-34 and remove its fully connection layers as the backbone of our network (He et al., 2016). Residual blocks in ResNet-34 use shortcuts to extract features more efficiently, transfer gradients and prevent gradients vanishing in deep layers. A residual block is consisted of convolution layers, batch normalization layers and rectified linear unit (ReLU), as shown in Figure 2.

**FIGURE 3:**
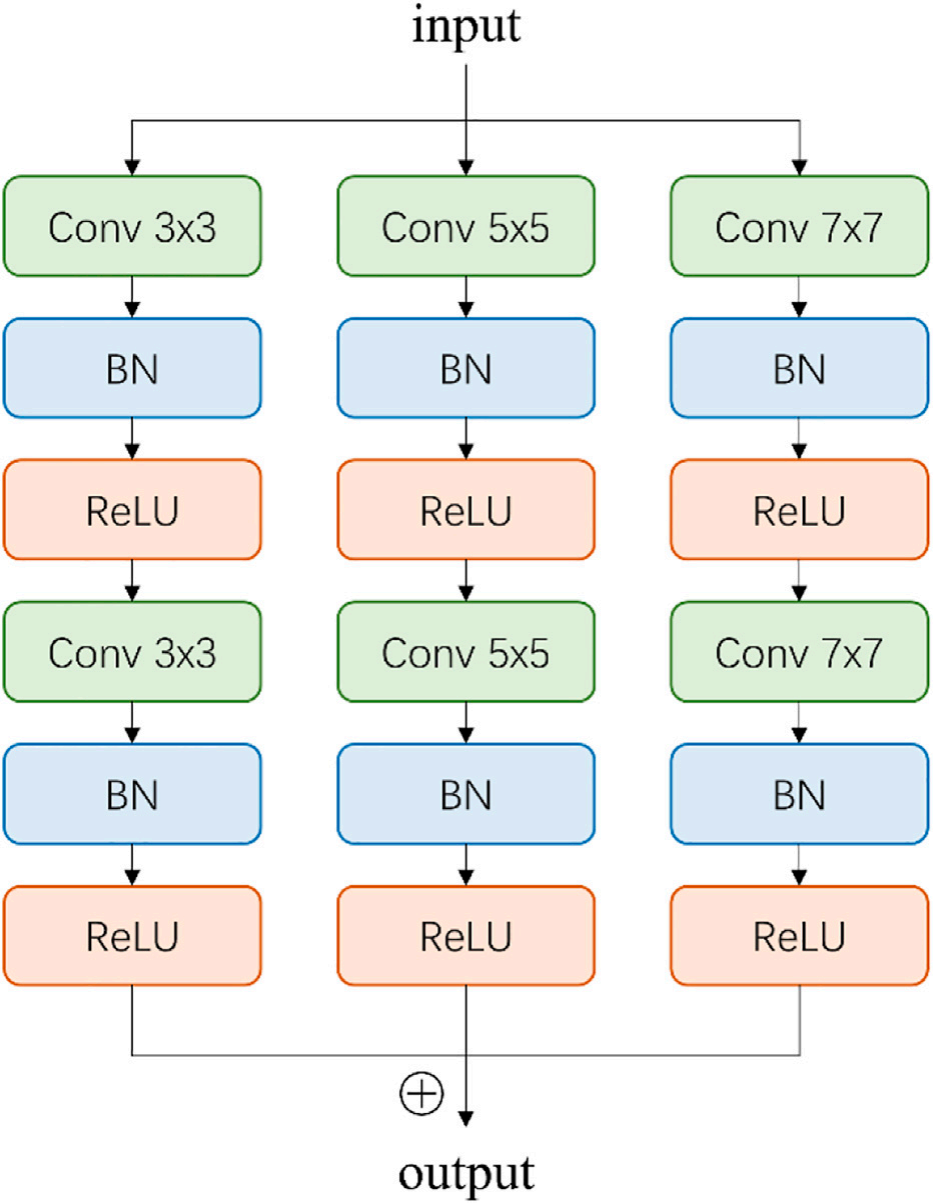
Illustration of multi-scale perception block (MSP). The 3 × 3, 5 × 5, and 7 × 7 convolution layers are realized by 3 × 3 dilatation convolution with dilated rate 1, 2, and 3, respectively.

Inspired by CE-Net (Zaiwang et al., 2019), we add a multi-scale perception block (MSP) between encoder and decoder, as shown in Figure 3. In order to save calculation cost, MSP realizes multi-scale convolution operation through dilated convolution, as shown in Figure 3. Convolution kernels of different sizes have different receptive fields, allowing more features to be captured. In this study, MSP contains 3 × 3, 5 × 5, and 7 × 7 convolution layers, which are realized by 3 × 3 dilatation convolution with dilated rate 1, 2, and 3, respectively. Finally, the feature graph is integrated by 1 × 1 convolution. After each convolutional layer, ReLU is added as activation function to increase the nonlinearity of the network.

In the decoder, each step consists of a bilinear interpolation operation, which is responsible for upscaling the feature map resolution by four times, followed by two convolutional blocks (Figure 2) and a concatenation with the corresponding feature maps from the encoder. At the final layer, a 1 × 1 padded convolutional layer was used to map the multi-channel feature maps to three classes that are belonged to eyelid region, MG region, and background region. The output of network was converted to a grayscale segmentation map, in which value 0, 1, and 2 are responding to background pixel, eyelid pixel, and MG region pixel, respectively.

### MG loss rate calculation

MG loss rate is the ratio of the area of meibomian gland loss to the total eyelid area. Therefore, the MG loss rate is a positive real number in the range [0,1]. The loss rate can be expressed by Eq. 1.

$$MG\;atrophy\;rate=1-\frac{MG\;area}{Eyelid\;area}$$ (1)

MG loss rate is a parameter directly related to the severity of MGD and is the most used in clinic. When the meibomian gland atrophied by 33%, the severity of MGD increased by one grade, which is known as meiboscore (Arita et al., 2014; Arita et al., 2008).

## Results

Two models were trained with the training set, including: 1) the model was trained with the k5m generation graph; 2) K5M images enhanced by CLAHE were used for training. Each training image and its corresponding mask were resized to 256 × 256 and augmented by gamma transformation, rotation, blur, noise addition, and image flip. The data augmentation increased the training images by 12 times. Training and evaluating of the proposed models were performed on a computer with an Nvidia GeForce GTX 3090 GPU. The deep learning models were implemented based on PyTorch (version 1.1.0) package in Python. All models were trained using the Adam optimizer (*α* = 0.9, *β* = 0.999), with an initial learning rate of 0.0003 and decays 0.8 times every five epochs. The batch size is set to 32 and the maximum epoch number of 50. L1 regulation was applied to prevent over-fitting (Hawkins, 2004). The loss function used in the experiment is Dice loss function (Jadon, 2020), which is represented by Eq. 2.

$$L_{Dice}=1-\frac{2\cdot \left|y\cap y^{\prime }\right|}{\left|y\right|+\left|y^{\prime }\right|}$$ (2)

To explore the influence of HS and CLAHE on model performance, k5m images and KH images were divided into different testing sets, which were: 1) $I_{k5m}$ and $I_{kh}$ ; 2) apply HS to KH image with $I_{k5m}$ as the guide images; 3) preprocessed $I_{k5m}$ and $I_{kh}$ using CLAHE. The specific division is shown in Table 1.

**TABLE 1:** Segmentation results of testing images from KH.

	MG region			Eyelid region		
	DSC	Recall	Precision	DSC	Recall	Precision
$I_{kh}$	0.70 ± 0.26	0.75 ± 0.29	0.67 ± 0.27	0.90 ± 0.10	0.89 ± 0.12	0.92 ± 0.07
$I_{kh-HS}$	0.69 ± 0.27	0.73 ± 0.29	0.67 ± 0.27	0.89 ± 0.10	0.88 ± 0.12	0.92 ± 0.08
$I_{kh-CLAHE}$	**0.71 ± 0.26**	**0.75 ± 0.27**	0.69 ± 0.26	0.90 ± 0.06	0.89 ± 0.10	**0.92 ± 0.07**
$I_{kh-HS-CLAHE}$	0.70 ± 0.25	0.72 ± 0.27	**0.71 ± 0.27**	**0.91 ± 0.05**	**0.91 ± 0.08**	0.91 ± 0.07

$I_{kh}$ : Testing images from KH without any preprocessing.

$I_{kh-HS}$ : Testing images from KH with HS.

$I_{kh-CLAHE}$ : Testing images from KH with CLAHE.

$I_{kh-HS-CLAHE}$ : Testing images from KH with HS and CLAHE.

Bold values are indicates the best performance.

The performance of the MG segmentation models was assessed by dice similarity coefficient (DSC), recall, and precision. These results were reported in Table 1 and Table 2. The results in Table 1 show that all images have close results. The maximum value of each indicator is marked in bold. Table 3 shows the comparison between segmentation results of different preprocessing methods on two image set. Note that there is no statistical difference in all different testing sets, except that the precision between the $I_{kh-CLAHE}$ and $I_{kh-HS-CLAHE}$ is statistically different. Similar results can also be found in the $I_{k5m}$ and $I_{k5m-CLAHE}$ , with no statistically significant difference between the evaluation results of the segmentation model regardless of whether the images were processed with CLAHE or not.

**TABLE 2:** Segmentation results of testing images from K5M.

	MG region			Eyelid region		
	DSC	Recall	Precision	DSC	Recall	Precision
$I_{k5m}$	**0.84 ± 0.11**	**0.79 ± 0.15**	**0.93 ± 0.07**	**0.92 ± 0.05**	**0.87 ± 0.08**	0.97 ± 0.08
$I_{k5m-CLAHE}$	**0.84 ± 0.11**	**0.79 ± 0.15**	0.92 ± 0.07	**0.92 ± 0.05**	**0.87 ± 0.08**	**0.98 ± 0.03**

$I_{k5m}$ : Testing images from K5M without any preprocessing.

$I_{k5m-CLAHE}$ : Testing images from K5M with CLAHE.

Bold values are indicates the best performance.

**TABLE 3:** Comparison between segmentation results of different preprocessing methods.

	MG region			Eyelid region		
	DSC	Recall	Precision	DSC	Recall	Precision
	*p*-value	*p*-value	*p*-value	*p*-value	*p*-value	*p*-value
$I_{kh}$ - $I_{kh-HS}$	0.36	0.12	0.16	0.42	0.16	0.41
$I_{kh}$ - $I_{kh-CLAHE}$	0.38	0.20	0.16	0.39	0.17	**0.0004**
$I_{k5m}$ - $I_{k5m-CLAHE}$	0.40	0.38	0.43	0.37	0.40	0.34

*p* < 0.01 is considered a statistical difference (Mann-Whitney U test).

The bold value here means that the *p* value less that 0.05 which indicates a statistical difference.

**FIGURE 4:**
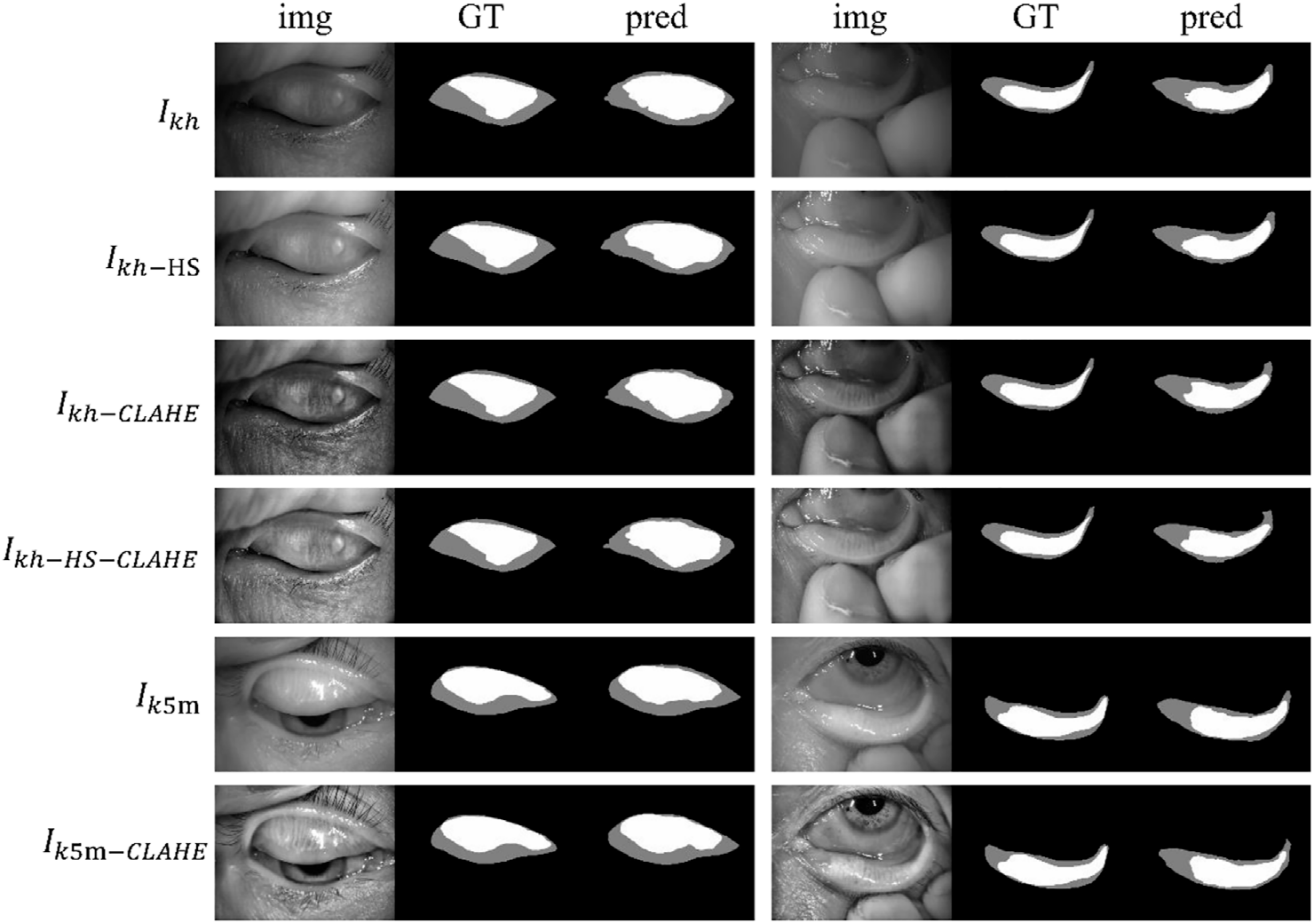
Examples of segmentation results for external and internal testing images. Img, GT, and pred represent input images, ground-truth masks, and predicted segmentation masks, respectively.

**FIGURE 5:**
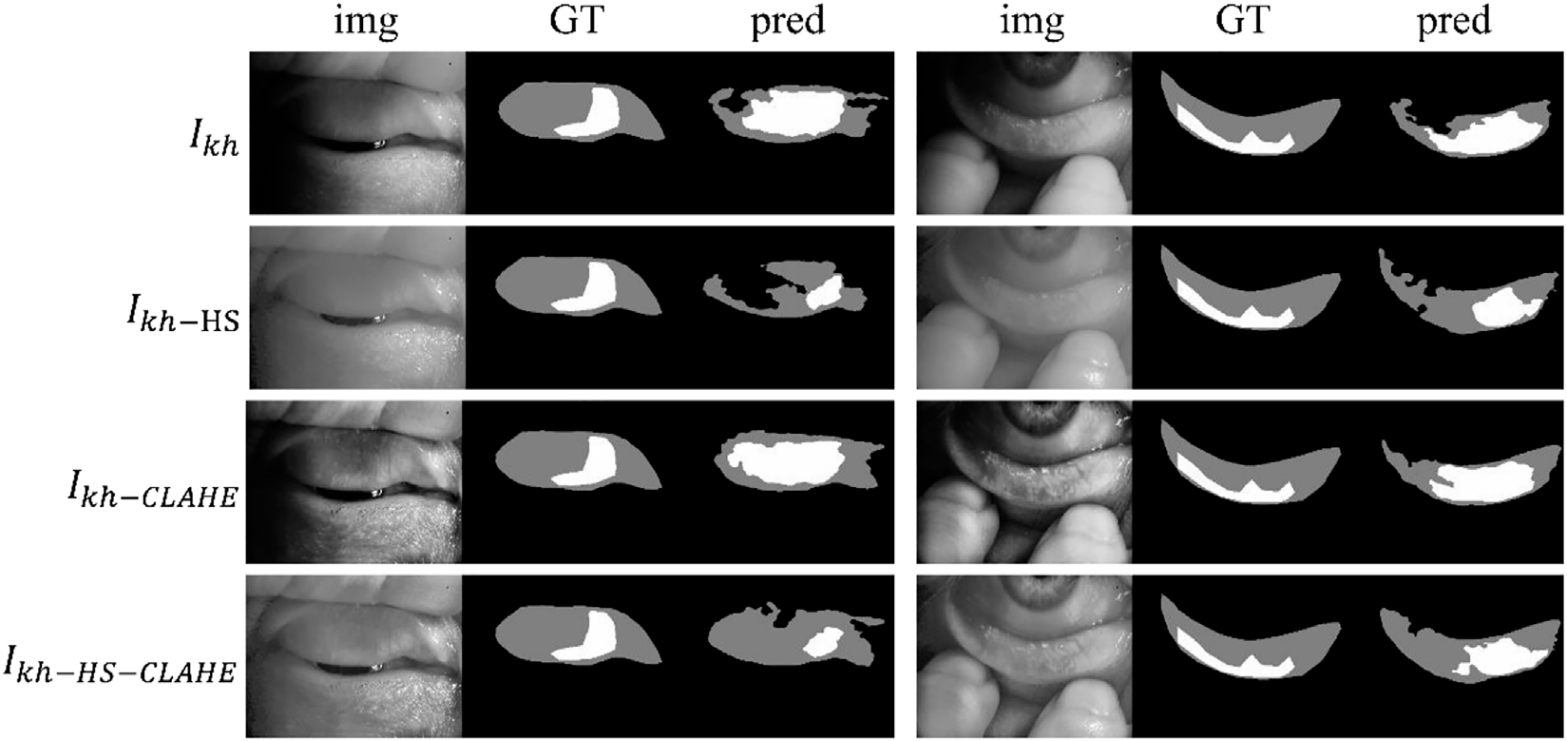
Some under-segmented examples in the external testing set due to the distribution gap. Img, GT, and pred represent input images, ground-truth masks, and predicted segmentation masks, respectively.

Figure 4 shows some visualization of MG segmentation results. Most of the images in both the internal testing set and the external testing set have segmentation results with high DSC score, and the network predicted segmentation masks and ground-truth masks have high similarity and coincidence. But some images in the external testing set have very low segmentation results, as shown in Figure 5, both the eyelid region and the MG region are under-segmented, with rough edges and anomalous masks. These under-segmented images have dissimilar features when compared with the predicted results on average level.

**FIGURE 6:**
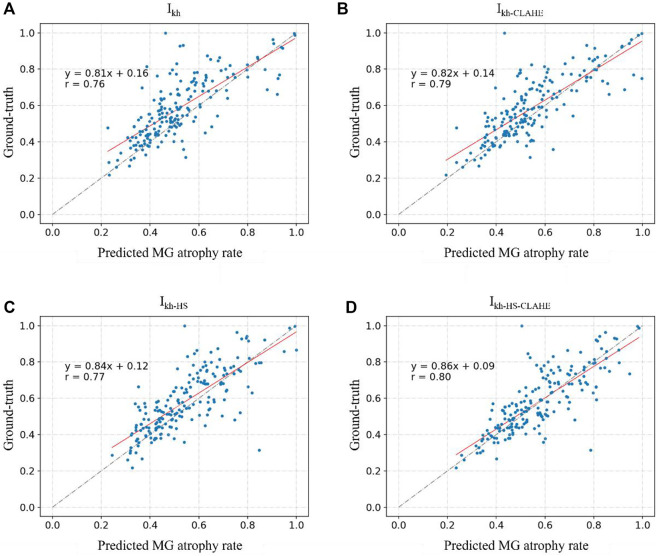
**(A–D)** represent the direct comparison of the ground truth and predicted MG atrophy rates of the unprocessed image set Ikh, image set Ikh-CLAHE processed with CLAHE, image set Ikh-HS processed with HS, and image set Ikh-HS-CLAHE processed with HS and CLAHE, respectively.

**FIGURE 7:**
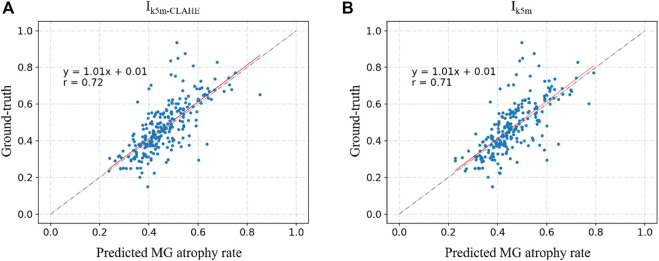
**(A)** and **(B)** represent the direct comparison of the ground real and predicted MG atrophy rates of the unprocessed image set Ik5m, image set I5m‐CLAHE processed with CLAHE, respectively.

Figure 6 and Figure 7 show the direct comparison results between the MG loss rate and GT for the external and internal testing sets. Most of the predicted MG loss rates are distributed near the ideal line (predicted MG loss rate equals to ground-truth MG loss rate), which indicates that the MG loss rates calculated according to the predicted segmentation results of the network are relatively close to the ground-truth. Root Mean Squared Error (RMSE) in Table 4 was used to quantify and compare the MG loss rate with the ground-truth. Testing set $I_{k5m-CLAHE}$ has the smallest RMSE when compared with ground-truth (RMSE = 0.09).

**TABLE 4:** RMSE of internal and external testing set compared with ground-truth.

	$I_{kh}$	$I_{kh-HS}$	$I_{kh-CLAHE}$	$I_{kh-HS-CLAHE}$	$I_{k5m}$	$I_{k5m-CLAHE}$
RMSE	0.12	0.12	0.13	0.11	0.10	**0.09**

The bold value here means the smallest RMSE and the best performance.

## Discussion

Meibomian gland morphology analysis is an important index to evaluate MGD. However, the current manual grading methods have the problems of heavy workload, low efficiency and large error, which is not conducive to the standardized diagnosis and treatment of MGD and dry eye. In this study, we developed and evaluated the automatic segmentation method of the eyelid area and the MG area based on CNN and automatically calculated MG loss rate. This method is evaluated in the internal and external testing sets from the two meibography devices. In addition, we also tested that the pre-processing method of HS and CLAHE as MG images has not improved significantly.

**FIGURE 8:**
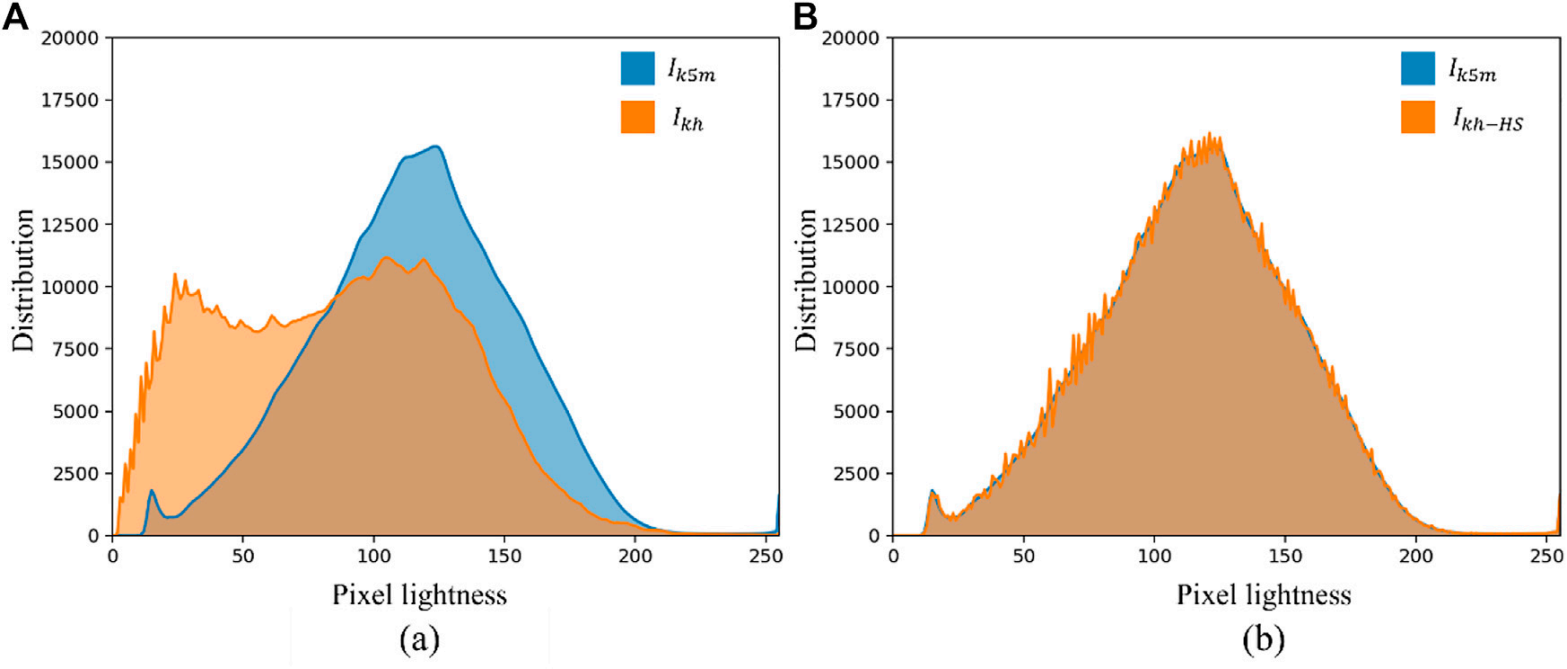
Histogram comparison of internal and external test sets. **(A)** Shows the histograms of $I_{kh}$ and $I_{k5m}$ ; **(B)** Shows the histograms of $I_{k5m}$ and $I_{kh-HS}$ .

**FIGURE 9:**
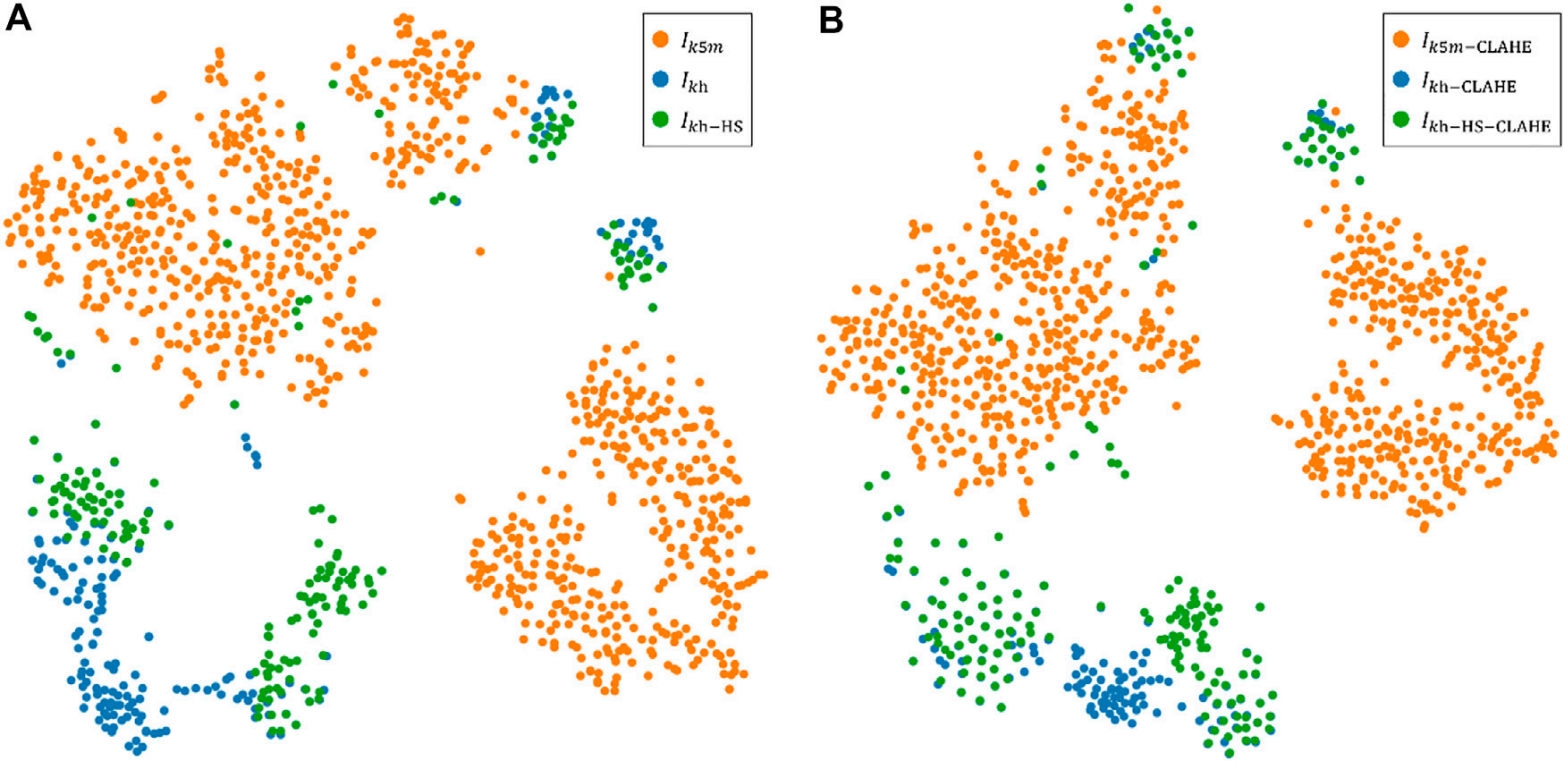
Execution of t-SNE algorithm for images from two different devices. Colors represent data from different devices. **(A)** A visualization of the t-SNE 2D non-linear embedding projection for the images without CLAHE. **(B)** A visualization of the t-SNE 2D non-linear embedding projection for the images with CLAHE for contrast enhancement.

In this experiment, the segmentation results of the external testing set ( $I_{kh}$ , $I_{kh-HS}$ , $I_{kh-CLAHE}$ , and $I_{kh-HS-CLAHE}$ ) from another device are lower than the internal testing set ( $I_{k5m}$ and $I_{k5m-CLAHE}$ ). Such performance gap is common in segmentation tasks based on CNN. Even though data augmentation was used in the training phase, data augmentation in the distribution domain of the training set ( $I_{k5m}$ and $I_{k5m-CLAHE}$ ) cannot close the distribution gap, because domain shift is systematic. As shown in the histogram in Figure 8, $I_{kh}$ and $I_{k5m}$ have very different pixel distributions. These image differences lead to a performance gap in the segmentation performance of the model between the two different testing sets. We used HS to make the histograms much more similar, but the obtained network segmentation results did not improve significantly. t-SNE (Van der Maaten and Hinton, 2008) was used to gain insight into the difference between the two sets of images before and after using HS, as shown in Figure 9A. The images are applied dimensionality reduction and projected onto a 2D plane. There is a large gap between the distributions of $I_{k5m}$ and $I_{kh}$ . Also, HS does not effectively reduce the distribution gap, although the histograms had been matched. Changing the low-dimensional image feature of gray distribution will not affect CNN to extract and learn the high-dimensional semantic information of the image.

CLAHE can effectively enhance the visual effect of MG images, which is beneficial for doctors to read and measure MG images manually. However, CLAHE does not significantly improve the segmentation results of neural networks, because CLAHE also fails to close the distribution gap between the distributions of $I_{k5m}$ and $I_{kh}$ , as shown in Figure 9B. CNN have been shown to be feasible in medical image segmentation tasks, but when used in practice, network models can suffer huge performance degradations if image data from other distributed domains are involved. Such performance gap can be explained using the independent and identically distributed (i.i.d.) assumption of statistical learning: a network model that is well-trained in the source distribution domain does not necessarily achieve similar high performance on datasets with the same distribution as the source distribution domain performance (Zhu et al., 2017). Adjusting the pixel distribution of images by HS and CLAHE did not improve the prediction results of the network model, although the images were visually very similar to the source distribution domain. Through tsne-2D visualization, it can be found that HS and CLAHE do not close the distribution gap.

## Conclusion

In this study, we developed and validated a CNN-based automatic MG analysis method based on MG images acquired by two different devices, K5M and KH. Predictions of MG loss rates were in high agreement with the gold standard obtained by physicians. We also found that in this study HS and CLAHE do not significantly improve the performance of CNN for segmenting MG, which may indicate that in future deep learning-based MG analysis tasks, no additional computational cost is required for image preprocessing phase.

## Data Availability

The raw data supporting the conclusion of this article will be made available by the authors, without undue reservation.

## References

[B1] Arita R. Itoh K. Inoue K. Amano S. (2008). Noncontact infrared meibography to document age-related changes of the meibomian glands in a normal population. Ophthalmology 115, 911–915. 10.1016/j.ophtha.2007.06.031 18452765

[B2] Arita R. Suehiro J. Haraguchi T. Shirakawa R. Tokoro H. Amano S. (2014). Objective image analysis of the meibomian gland area. Br. J. Ophthalmol. 98, 746–755. 10.1136/bjophthalmol-2012-303014 23813417 PMC4033206

[B3] Celik T. Lee H. K. Petznick A. Tong L. (2013). Bioimage informatics approach to automated meibomian gland analysis in infrared images of meibography. J. Optometry 6, 194–204. 10.1016/j.optom.2013.09.001

[B4] da Cruz L. B. Araújo J. D. L. Ferreira J. L. Diniz J. O. B. Silva A. C. de Almeida J. D. S. (2020). Kidney segmentation from computed tomography images using deep neural network. Comput. Biol. Med. 123, 103906. 10.1016/j.compbiomed.2020.103906 32768047

[B5] Dai Q. Liu X. Lin X. Fu Y. Chen C. Yu X. (2021). A novel meibomian gland morphology analytic system based on a convolutional neural network. IEEE Access 9, 23083–23094. 10.1109/ACCESS.2021.3056234

[B6] Den S. Danielnelson J. Mcculley J. P. Shimazaki J. Craig J. Benitez-Del-Castillo J. M. (2011). The international workshop on meibomian gland dysfunction: Report of the definition and classification subcommittee. Invest. Ophthalmol. Vis. Sci. 52, 1930–1937. 10.1167/iovs.10-6997b 21450914 PMC3072158

[B7] Djalilian A. R. (2018). “Ocular surface disease,” in Update in the diagnosis and management of meibomian gland dysfunction (Berlin, Germany: Springer), 17–29. 10.1007/978-3-319-15823-5

[B8] Driver P. J. Lemp M. A. (1996). Meibomian gland dysfunction. Surv. Ophthalmol. 40, 343–367. 10.1016/s0039-6257(96)80064-6 8779082

[B9] Erich K. Nadja K. Thomas M. Hiroto O. Sullivan D. A. (2011). The international workshop on meibomian gland dysfunction: Report of the subcommittee on anatomy, physiology, and pathophysiology of the meibomian gland. Invest. Ophthalmol. Vis. Sci. 52, 1938–1978. 10.1167/iovs.10-6997c 21450915 PMC3072159

[B10] Hawkins D. M. (2004). The problem of overfitting. J. Chem. Inf. Comput. Sci. 44, 1–12. 10.1021/ci0342472 14741005

[B11] He K. Zhang X. Ren S. Sun J. (2016). “Deep residual learning for image recognition,” in Proceedings of the IEEE Conference on Computer Vision and Pattern Recognition, Las Vegas, NV, USA, 27-30 June 2016, 770–778.

[B12] Hwang H. S. Mikula E. Xie Y. Brown D. J. Jester J. V. (2021). A novel transillumination meibography device for *in vivo* imaging of mouse meibomian glands. Ocul. Surf. 19, 201–209. 10.1016/j.jtos.2020.08.012 33075493 PMC10388835

[B13] Jadon S., 2020. A survey of loss functions for semantic segmentation. arXiv preprint arXiv:2006.14822.

[B14] Jo J. Bengio Y., 2017. Measuring the tendency of cnns to learn surface statistical regularities. arXiv preprint arXiv:1711.11561.

[B15] Koprowski R. Tian L. Olczyk P. (2017). A clinical utility assessment of the automatic measurement method of the quality of Meibomian glands. Biomed. Eng. Online 16, 82. 10.1186/s12938-017-0373-4 28646862 PMC5483265

[B16] Koprowski R. Wilczyński S. Olczyk P. Nowińska A. Węglarz B. Wylęgała E. (2016). A quantitative method for assessing the quality of meibomian glands. Comput. Biol. Med. 75, 130–138. 10.1016/j.compbiomed.2016.06.001 27286185

[B17] Liang F. Xu Y. Li W. Ning X. Liu X. Liu A. (2017). Recognition algorithm based on improved FCM and rough sets for meibomian gland morphology. Appl. Sci. 7, 192. 10.3390/app7020192

[B18] Llorens-Quintana C. Rico-del-Viejo L. Syga P. Madrid-Costa D. Iskander D. R. (2019). A novel automated approach for infrared-based assessment of meibomian gland morphology. Trans. Vis. Sci. Tech. 8, 17. 10.1167/tvst.8.4.17 PMC6681863 31392084

[B19] McDonald M. Patel D. A. Keith M. S. Snedecor S. J. (2016). Economic and humanistic burden of dry eye disease in europe, north America, and asia: A systematic literature review. Ocul. Surf. 14, 144–167. 10.1016/j.jtos.2015.11.002 26733111

[B20] Naseem R. Khan Z. A. Satpute N. Beghdadi A. Cheikh F. A. Olivares J. (2021). Cross-modality guided contrast enhancement for improved liver tumor image segmentation. IEEE Access 9, 118154–118167. 10.1109/ACCESS.2021.3107473

[B21] Nichols K. K. Foulks G. N. Bron A. J. Glasgow B. J. Dogru M. Tsubota K. (2011). The international workshop on meibomian gland dysfunction: Executive summary. Invest. Ophthalmol. Vis. Sci. 52, 1922–1929. 10.1167/iovs.10-6997a 21450913 PMC3072157

[B22] Perone C. S. Ballester P. Barros R. C. Cohen-Adad J. (2019). Unsupervised domain adaptation for medical imaging segmentation with self-ensembling. NeuroImage 194, 1–11. 10.1016/j.neuroimage.2019.03.026 30898655

[B23] Prabhu S. M. Chakiat A. S S. Vunnava K. P. Shetty R. (2020). Deep learning segmentation and quantification of Meibomian glands. Biomed. Signal Process. Control 57, 101776. 10.1016/j.bspc.2019.101776

[B24] Pult H. Nichols J. J. (2012). A review of meibography. Optom. Vis. Sci. 89, E760–E769. 10.1097/OPX.0b013e3182512ac1 22488268

[B25] Pult H. Riede-Pult B. (2013). Comparison of subjective grading and objective assessment in meibography. Cont. Lens Anterior Eye 36, 22–27. 10.1016/j.clae.2012.10.074 23108007

[B26] Pult H. Riede-Pult B. H. (2012). Non-contact meibography: Keep it simple but effective. Cont. Lens Anterior Eye 35, 77–80. 10.1016/j.clae.2011.08.003 21885325

[B27] Ronneberger O. Fischer P. Brox T. (2015). “U-net: Convolutional networks for biomedical image segmentation,” in International Conference on Medical Image Computing and Computer-Assisted Intervention, Berlin, Germany, 18 November 2015 (Springer), 234–241.

[B28] Turgut B. Çatak O. Demir T. (2018). Meibomian gland dysfunction: An overlooked eyelid disease. Adv. Ophthalmol. Vis. Syst. 8. 10.15406/aovs.2018.08.00295

[B29] Van der Maaten L. Hinton G. (2008). Visualizing non-metric similarities in multiple maps. Mach. Learn. 9, 33–55. 10.1007/s10994-011-5273-4

[B30] Wang J. Yeh T. N. Chakraborty R. Yu S. X. Lin M. C. (2019). A deep learning approach for meibomian gland atrophy evaluation in meibography images. Trans. Vis. Sci. Tech. 8, 37. 10.1167/tvst.8.6.37 PMC6922272 31867138

[B31] Wong S. Srinivasan S. Murphy P. J. Jones L. (2019). Comparison of meibomian gland dropout using two infrared imaging devices. Cont. Lens Anterior Eye 42, 311–317. 10.1016/j.clae.2018.10.014 30413376

[B32] Xiao B. Tang H. Jiang Y. Li W. Wang G. (2018). Brightness and contrast controllable image enhancement based on histogram specification. Neurocomputing 275, 2798–2809. 10.1016/j.neucom.2017.11.057

[B33] Xiao J. Adil M. Y. Chen X. Utheim Ø. A. Ræder S. Tønseth K. A. (2020). Functional and morphological evaluation of meibomian glands in the assessment of meibomian gland dysfunction subtype and severity. Am. J. Ophthalmol. 209, 160–167. 10.1016/j.ajo.2019.09.005 31526799

[B34] Xiao P. Luo Z. Deng Y. Wang G. Yuan J. (2021). An automated and multiparametric algorithm for objective analysis of meibography images. Quant. Imaging Med. Surg. 11, 1586–1599. 10.21037/qims-20-611 33816193 PMC7930676

[B35] Yan W. Wang Y. Gu S. Huang L. Yan F. Xia L. (2019). “The domain shift problem of medical image segmentation and vendor-adaptation by unet-GAN,” in Medical image computing and computer assisted intervention – MICCAI 2019, lecture notes in computer science. Editors Shen D. Liu T. Peters T. M. Staib L. H. Essert C. Zhou S. (Cham: Springer International Publishing), 623–631. 10.1007/978-3-030-32245-8_69

[B36] Zaiwang G. Jun C. Huazhu F. Kang Z. Huaying H. Yitian Z. 2019, CE-Net: Context encoder network for 2D medical image segmentation. arXiv:1903.02740v1. 10.1109/TMI.2019.2903562 30843824

[B37] Zhu J.-Y. Park T. Isola P. Efros A. A. (2017). “Unpaired image-to-image translation using cycle-consistent adversarial networks,” in Proceedings of the IEEE International Conference on Computer Vision, Venice, Italy, 22-29 October 2017, 2223–2232.

